# Trehalose can effectively protect sheep epididymis epithelial cells from oxidative stress

**DOI:** 10.5194/aab-64-335-2021

**Published:** 2021-08-18

**Authors:** Zhaojin Luan, Xiaomei Fan, Yongchao Zhao, Huizi Song, Wei Du, Jiaoxia Xu, Zhaochen Wang, Wenguang Zhang, Jiaxin Zhang

**Affiliations:** 1 College of Animal Science, Inner Mongolia Agricultural University, Hohhot, Inner Mongolia, 010018, China; 2 Inner Mongolia Key Laboratory of Animal Genetics, Breeding and Reproduction, Inner Mongolia Agricultural University, Hohhot, Inner Mongolia, 010018, China; 3 Baotou Medical College, Baotou, Inner Mongolia, 014040, China; 4 Basic Medical College, Inner Mongolia Medical University, Hohhot 010059, Inner Mongolia, China

## Abstract

Trehalose, a naturally nontoxic disaccharide that does not exist in mammals, stabilizes cell membrane integrity under oxidative stress conditions, the mechanism of which is still unclear. Here, we analyzed the effects of trehalose on sheep epididymis epithelial cell (EEC) proliferation and its possible mechanisms. To study the effect of trehalose on EECs, EECs were isolated from testes of 12-month-old sheep; cell counting kit-8 (CCK-8) was used to measure the growth of the cells. Cell proliferation was evaluated by assaying cell cycle and apoptosis, and RT-PCR was utilized to identify the epididymal molecular markers glutathione peroxidase 5 (*GPX5*) and androgen receptor (*AR*). Next, reactive oxygen species (ROS) content was evaluated by a dichloro-dihydro-fluorescein diacetate (DCFH-DA) probe. Superoxide dismutase (SOD), catalase (CAT), and glutathione peroxidase (GSH-Px) activities were evaluated by enzyme chemistry methods, and *GPX5* expression was evaluated by qRT-PCR and enzyme-linked immunosorbent assay (ELISA). The results showed that 100 mM trehalose significantly improved the proliferation potential of EECs, in which the cells could be serially passaged 14 times with continued normal *GPX5* and *AR* marker gene expression in vitro. The trehalose can increase significantly a proportion of EECs in S phase (P<0.01) and decrease significantly the apoptotic rate of EECs (P<0.01) compared to the control. Moreover, the trehalose decreased ROS significantly (P<0.01) and increased CAT (P<0.01) and GSH-Px (P<0.05) activities significantly in EECs. *GPX5* mRNA and protein expression were also significantly upregulated in trehalose-treated EECs (P<0.05 and P<0.01 respectively). Our study suggested that exogenous trehalose exhibited antioxidant activity through increasing the activities of CAT, GSH-Px, and the expression level of *GPX5* and could be employed to maintain vitality of sheep EECs during long-term in vitro culture.

## 1 Introduction

The epididymis is an important male reproductive organ in mammals for sperm maturation (Qu et al., 2014). The caput of the epididymis is crucial for the early and late processes of spermatozoa maturation (Leir et al., 2015). Sperm mature and acquire motility as they interact with proteins synthesized and secreted by epididymal epithelial cells (EECs) during passage through the epididymal lumen (Cornwall, 2009). Primary cells can help elucidate the molecular mechanisms of epididymis function in vitro (Leir et al., 2015). The expression patterns of many functional genes in epididymis are species-specific (Jalkanen et al., 2006; Thimon et al., 2007), which makes it necessary to establish different animal primary EEC lines.

Reactive oxygen species (ROS) were produced during redox metabolism in cells, and appropriate amounts of ROS directly interact with critical signaling molecules to initiate a broad variety of cellular processes, including cell proliferation and survival (Ray et al., 2012; Öztürk et al., 2019). However, excessive ROS can cause oxidative stress and cell damage, which hinders cell growth (Rhee, 1999). In the process of cell culture in vitro, cells need to avoid oxidative stress in order to maintain their normal function.

Trehalose is a natural, nonpermeable disaccharide with the unique ability to stabilize and protect the structure of proteins, viruses, and bacteria, as well as to maintain the cell homeostasis and handle various stress conditions (Rossi et al., 1997; Singer and Lindquist, 1998; Bieganski et al., 1998; Elbein, 2003; Öztürk et al., 2019). Trehalose has been employed in tissue preservation (Hirata et al., 1994). Previous studies have shown that trehalose could be used as an additive in cell culture to significantly improve the survival rate of human hematopoietic cells, human embryonic kidney cells, mammalian keratinocytes, and fibroblasts during cryopreservation (Eroglu et al., 2000; Limaye and Kale, 2001; Sasnoor et al., 2005; Hara et al., 2017). Although trehalose can maintain cell viability under stress conditions, the effect of trehalose on sheep EECs in vitro still is not clear.

In recent years, studies have shown that trehalose protects cells from damage by oxygen free radicals (Luyckx and Baudouin, 2011; Büyükleblebici, 2014; Bucak et al., 2021). Yeast cells exposed to a free radical-generating system were found to accumulate trehalose (Nishimoto et al., 2016). Arsenite can induce the production of ROS by *Candida albicans*, stimulating the accumulation of trehalose and the activation of catalase in the cells (Urrialde et al., 2017). Trehalose pretreatment effectively prevents the oxidative damage of yeast cells by superoxide (Herdeiro et al., 2006; Uysal et al., 2009; Bucak et al., 2020; Öztürk et al., 2020). Trehalose also protects sperm from oxidative stress and maintains the integrity of the sperm acrosome membrane and plasma membrane (Hu et al., 2010).

This study investigated the effect of trehalose on the proliferation of sheep EECs in vitro and explored whether trehalose played an antioxidant role in these cell lines or not.

**Table 1:** Sequence of primers used for RT-PCR.

Gene	Sequence	Amplicon size (bp)	$T_{m}$ (${}^{\circ }C$)
*GPX5*	F:5${}^{\prime }$GCAATCCTGTCCTCACCCTT 3${}^{\prime }$R:5${}^{\prime }$ACGCCATCAGGTCCCACT 3${}^{\prime }$	119	60
*AR*	F:5${}^{\prime }$GACCATGTTCTGCCCATCGACTAT 3${}^{\prime }$R:5${}^{\prime }$CTGCTGGCACACAGGTACTTCTGT 3${}^{\prime }$	189	60
β*-actin*	F:5${}^{\prime }$GTCATCACCATCGGCAATGA 3${}^{\prime }$R:5${}^{\prime }$CGTGAATGCCGCAGGATT 3${}^{\prime }$	88	60

$T_{m}$, melting temperature.

## 2 Materials and methods

### 2.1 Ethics statement and sample collection

The protocols using animals in our study were approved by the Ethics Committee of Experimental Animal of Inner Mongolia Agricultural University, Inner Mongolia Autonomous Region, China (no. XMXK20180016). Testicles from three 12-month-old sheep, a local breed, were obtained from a slaughterhouse (Hohhot), immediately stored in phosphate-buffered saline (PBS) containing 100 $U\;{\mathrm{mL}}^{-1}$ penicillin and 100 µg ${\mathrm{mL}}^{-1}$ streptomycin (15070063, Gibco, Carlsbad, CA, USA) and 0.25 µg ${\mathrm{mL}}^{-1}$ amphotericin B (15290026, Gibco), and then stored in a 4 ${}^{\circ }C$ portable refrigerator and brought to the laboratory within 2 h.

### 2.2 Isolation and culture of primary cells

EECs were cultured following a modified protocol from Luan et al. (2019). Briefly, the caput epididymis was isolated from testis, fat, and connective tissue and washed in Dulbecco's PBS (Gibco) to remove blood. For tubule isolation, tissue was minced into small pieces (∼2–3 mm) using scissors and digested in 0.25 % trypsin-EDTA (25200056, Gibco) for 30 min at 37 ${}^{\circ }C$ in a water bath (shaking every 5–10 min). Digestion was terminated by the addition of serum, and the small tubules were isolated by gravity sedimentation. The tubules were then placed into a new 50 mL centrifuge tube with 25 mL digestion buffer containing 0.1 % type IV collagenase containing 0.36 mM ${\mathrm{CaCl}}_{2}$ and digested for 40 min at 37 ${}^{\circ }C$ in a water bath (shaking every 5–10 min) and filtered through a 70 µm nylon membrane. Finally, cells were pelleted by centrifugation at 600×g for 5 min, washed, and resuspended in a RPMI-1640 medium (11875093, Gibco) supplemented with 10 % fetal calf serum (10099141C, Gibco), 100 $\mathrm{nmol}\;L^{-1}$ testosterone (T6147, Sigma, St. Louis, MO, USA), 200 $\mathrm{nmol}\;L^{-1}$ hydrocortisone (H0888, Sigma), 5 µg ${\mathrm{mL}}^{-1}$ transferrin (11107047, Gibco), 10 $\mathrm{mg}\;{\mathrm{mL}}^{-1}$ insulin (I1507, Sigma), and 50 $U\;{\mathrm{mL}}^{-1}$ penicillin, and 50 µg ${\mathrm{mL}}^{-1}$ streptomycin. In the first week of culture, 0.25 µg ${\mathrm{mL}}^{-1}$ amphotericin B was added to the culture medium. Subsequently, cells were cultured in media without amphotericin B. Cells were seeded at $1\times 10^{4}$ $\mathrm{cells}\;{\mathrm{mL}}^{-1}$ in 60 mm petri dishes (Corning Incorporated, New York, NY, USA) and incubated at 37 ${}^{\circ }C$ with 5 % ${\mathrm{CO}}_{2}$. EECs that were not adherent 30 min after plating were transferred to another 60 mm petri dish, and the adherent fibroblasts could be abscised. The culture medium was changed every other day. The cells were passaged with 0.25 % trypsin-EDTA to amplify before they reached 80 % confluence, and digestion was terminated by the addition of serum. The morphology of cells was observed using a fluorescence microscope (Zeiss, Observer A1, Munich, Germany). Trehalose (T0167, Sigma) was fully dissolved in the above medium to stimulate cells. The final concentrations of 0, 50, 100, and 200 mM trehalose were used for growth curve detection, and 0 and 100 mM trehalose were used for cell cycle, apoptosis, ROS content, enzyme activity, and *GPX5* expression detection, respectively.

### 2.3 Immunofluorescence identification of EECs

The cells (passage 1) were cultured in a one-chamber slide (Nalge Nunc, Rochester, NY, USA) at 37 ${}^{\circ }C$, fixed in 4 % paraformaldehyde for 30 min. The fixed cells were washed with PBS and permeabilized with PBS (pH 7.2) containing 0.3 % (v/v) Triton X-100 for 10 min at room temperature. The slides were blocked with 5 % BSA (A1933, Sigma) in PBS for 20 min at 37 ${}^{\circ }C$, washed with PBS, then incubated with mouse monoclonal Anti-Cytokeratin Peptide 18 antibody (ab82254, Abcam, Cambridge, MA, USA) at 1 : 100 dilution for 12 h at 4 ${}^{\circ }C$. The secondary antibody employed was FITC rabbit anti-mouse IgG secondary antibody (Bioss, Woburn, MA, USA) at 1 : 300 dilution for 1 h at 37 ${}^{\circ }C$. Fibroblasts were used as a control. Samples were examined with a fluorescence microscope (Zeiss, Imager A2, Munich, Germany).

### 2.4 Cell growth curve

Cells (2500 per well at passage 4) were seeded at a density of $2.5\times 10^{4}$ ${\mathrm{mL}}^{-1}$ in a 96-well plate with a 100 µL culture medium per well and cultured at 37 ${}^{\circ }C$ with 5 % ${\mathrm{CO}}_{2}$ for 12 h. The media were replaced and supplemented with 50, 100, or 200 mM trehalose in the respective treatment groups. A trehalose-free medium was used as a control. Media were replenished every 24 h. The cell proliferation rates on days 1–6 were subsequently assessed using the cell counting kit-8 (CCK-8) (FC101, TransGen Biotech, Beijing, China) according to the manufacturer's instructions. The absorbance at 450 nm was measured using a BioTek microplate reader (Winooski, VT, USA).

### 2.5 Reverse transcription (RT) polymerase chain reaction (PCR)

To measure whether different passage cells can maintain normal functional characteristics, EECs were cultured with 100 mM trehalose at passage (P)0, P1, P6, and P14 and cultured without trehalose at passage P0. Total RNA was isolated using TRIzol reagent (Tiangen, Beijing, China), and cDNAs were synthesized using the PrimeScript™ RT Master Mix (RR036A, TaKaRa, Dalian, China) according to the manufacturer's instructions. Glutathione peroxidase 5 (*GPX5*) and androgen receptor (*AR*) genes were PCR-amplified using Platinum™ II Hot-Start Green PCR Master Mix (2X) (14001012, Invitrogen) with β *-actin* as a control. PCR products were separated on a 1.5 % agarose gel, and a Fluor-s multi-imaging densitometer was used to observe the PCR products visualized with ethidium bromide. The design of primers refers to gene sequences in GenBank. Primers for amplification and melting temperatures are listed in Table 1.

### 2.6 Cell cycle distribution assay

The cells (passage 4) treated with 0 or 100 mM trehalose for 24 h were harvested using 0.25 % trypsin, washed with PBS three times, and then fixed at 4 ${}^{\circ }C$ for 12 h with 70 % ethanol. Fixed cells were subsequently rehydrated with PBS and incubated with 100 µL RNase A at 37 ${}^{\circ }C$ for 30 min. For staining of nuclei, cells were incubated with 400 µL propidium iodide (PI) solution for 30 min at 4 ${}^{\circ }C$ in the dark (CA1510, Solarbio, Beijing, China). Stained cells were subsequently analyzed using a flow cytometer (NovoCyte 1040, ACEA Biosciences Inc., CA, USA). For each analysis, 10 000 events were evaluated. Percentages of cells in G1, S, and G2 phases were directly obtained from dot plots and presented in bar charts.

**Figure 1:**
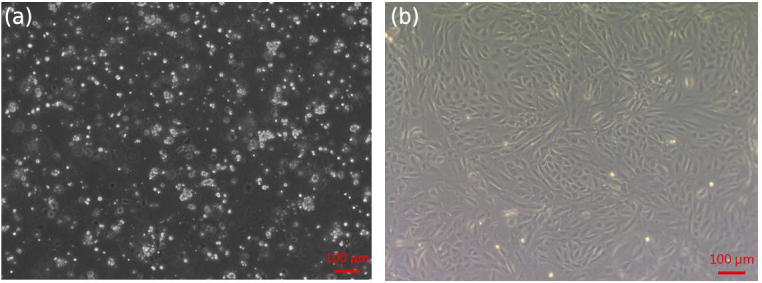
Isolation of sheep EECs. **(a)** Primary cells isolated from the epididymis of 12-month-old sheep. **(b)** First-passage monolayer cells reached confluency after 2–3 d of culture.

**Figure 2:**
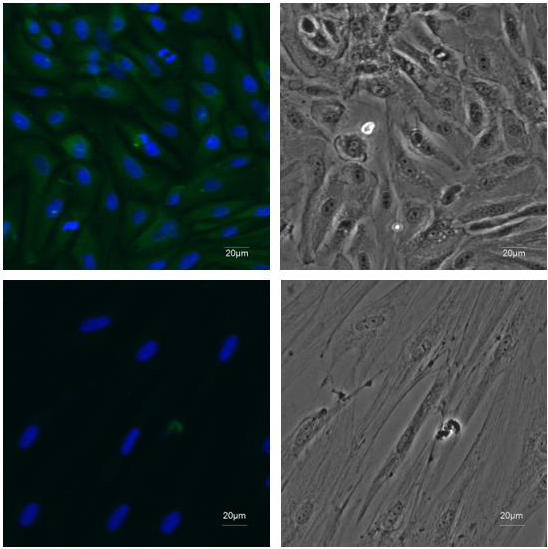
Identification of EECs. ×20 immunofluorescence was performed using antibodies recognizing CK18 (green) and DAPI to mark nuclei (blue) in the first-passage EECs. Fibroblasts were stained as negative control.

**Figure 3:**
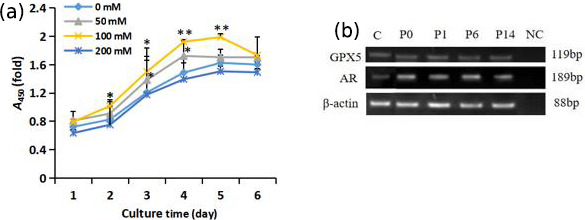
Growth of trehalose-treated sheep EECs. **(a)** The growth curve of sheep EECs treated with different concentrations of trehalose. * P<0.05 and ** P<0.01 compared with the 0 mM trehalose-treated group. Error bars indicate the standard deviations. **(b)** RT-PCR analysis of *GPX5* and *AR* in isolated EECs cultured with trehalose at passage (P)0, P1, P6, and P14. C refers to EECs cultured without trehalose at passage P0. NC refers to negative control, and β-*actin* was used as an internal control.

**Figure 4:**
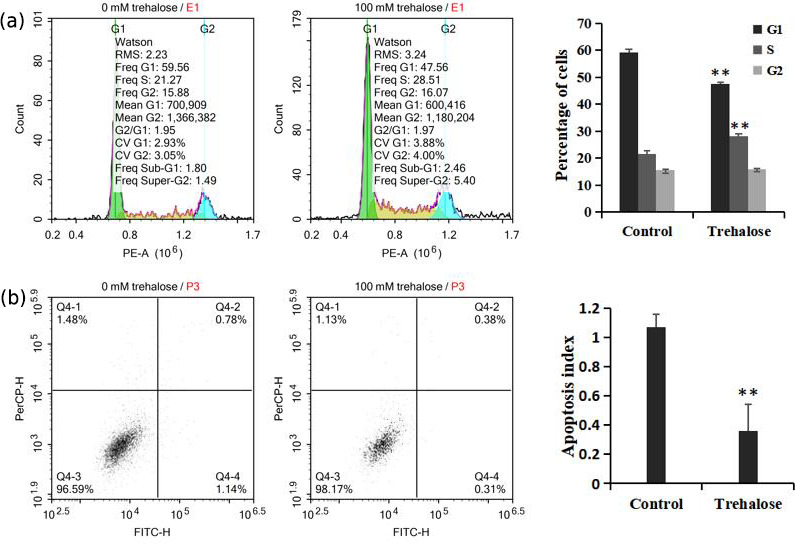
Effect of trehalose on cell cycle distribution and apoptosis of sheep EECs. The EECs were treated with trehalose (0 or 100 mM) for 24 h. **(a)** The cell cycle distribution in trehalose-free culture and 100 mM trehalose culture. **(b)** Apoptosis rate of cells cultured without trehalose and with 100 mM trehalose. * P<0.05 and ** P<0.01 vs. the trehalose-free group.

**Figure 5:**
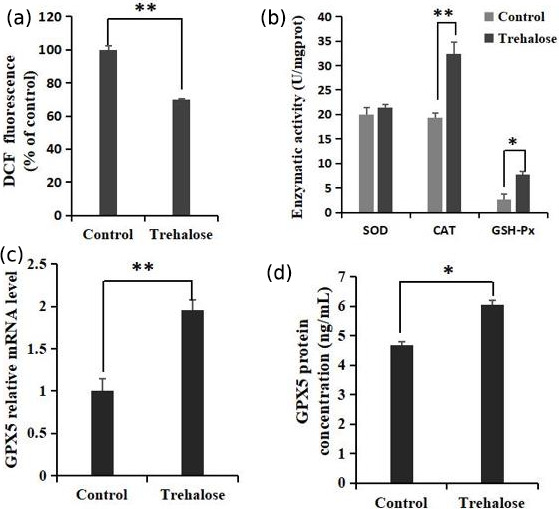
Effects of trehalose on ROS and antioxidant enzymes in sheep EECs. **(a)** The EECs were loaded with DCFH-DA (10 µM) to capture the ROS-generated fluorescence after trehalose (100 mM) pretreatment for 24 h. The DCF fluorescence is expressed as % of control. **(b)** Activities of SOD, CAT, and GSH-Px in EECs treated with trehalose (100 mM). **(c)** The expression of *GPX5* mRNA was detected by qRT-PCR. **(d)** The expression of GPX5 protein was detected by ELISA. Control was EECs with 0 mM trehalose treatment. * P<0.05 and ** P<0.01.

### 2.7 Apoptosis assay

Cells (passage 4) treated with 0 or 100 mM trehalose for 24 h were collected with 0.25 % trypsin, washed with PBS, and resuspended in 1× binding buffer with a concentration of $1\times 10^{6}$ cells ${\mathrm{mL}}^{-1}$ incubated at room temperature for 15 min in the dark with 5 µL Annexin VFITC and 3 µL PI solution (FITC Annexin V Apoptosis Detection Kit1, BD Biosciences, CA, USA). The samples were then measured using a flow cytometer (NovoCyte 1040, ACEA Biosciences Inc.). Apoptotic cells were defined as Annexin V+ PI-.

### 2.8 Measurement of ROS

ROS content in the cells was tested using a dichloro-dihydro-fluorescein diacetate (DCFH-DA) fluorescent dye (Beyotime, Shanghai, China). Briefly, after 0 or 100 mM trehalose treatment for 24 h, cells (passage 4) were incubated with 10 µM DCFH-DA at room temperature for 30 min and then analyzed for fluorescence via a flow cytometer (NovoCyte 1040, ACEA Biosciences Inc.). ROS intensity in the treatment group was expressed as fold change of that in the control group.

### 2.9 Assay of SOD, CAT, and GSH-Px activities

The enzymatic activities were all determined by assay kits (Beyotime). For the determination of superoxide dismutase (SOD) activity, the 2-(4-iodophenyl)-3-(4-nitrophenyl)-5-(2,4-disulfophenyl)-2H-tetrazolium (WST-1) method was used, where in the SOD activity can be calculated by measuring the absorbance of formazan dye at 450 nm. The assay for catalase (CAT) was based on its ability to scavenge $H_{2}O_{2}$. Glutathione peroxidase (GSH-Px) catalyst activity was assayed by quantifying the rate of oxidation of the reduced glutathione to the oxidized glutathione by $H_{2}O_{2}$. As such, cells ($1\times 10^{6}$ ${\mathrm{mL}}^{-1}$ at passage 4) were plated in 60 mm petri dishes. After 0 or 100 mM trehalose treatment for 24 h, cells were washed twice in ice-cold PBS and homogenized. The homogenate was centrifuged for 10 min at 10 000 rpm at 4 ${}^{\circ }C$, and supernatant was used for determining SOD, CAT, and GSH-Px activities according to the manufacturer's instructions.

### 2.10 Quantitative RT-PCR

To characterize *GPX5* gene expression by quantitative (q)RT-PCR, passage-4 cells, treated with 0 or 100 mM trehalose for 24 h, were extracted using TRIzol reagent (Tiangen). cDNA was synthesized using PrimeScript™ RT Master Mix (RR036A, TaKaRa) according to the manufacturer's instructions. Quantification of expressions of *GPX5* and β *-actin* mRNAs was determined using SYBR Premix Ex Taq™ II (RR820A, TaKaRa) according to the manufacturer's instructions. Data were analyzed by the 2${}^{-\Delta \Delta ct}$ method. Primers for amplification and melting temperatures are listed in Table 1.

### 2.11 Detection of protein expression by ELISA

Enzyme-linked immunosorbent assay (ELISA) was performed using sheep GPX5 ELISA kits from Bowman Biotech (Shanghai, China) to detect GPX5 protein expression. Briefly, cells (passage 4) treated with 0 or 100 mM trehalose for 24 h were treated with 200 µL protein extraction reagent (78503, Thermo Scientific, USA). A standard curve was created by using 50 µL of each standard. Sample wells contained 10 µL sample and 40 µL diluent. The cells in standard and sample wells were incubated with 100 µL HRP-labeled detection antibody at 37 ${}^{\circ }C$ for 60 min and with 50 µL A and B substrates at 37 ${}^{\circ }C$ for 15 min in the dark. The absorbance at 450 nm was measured using a BioTek microplate reader (Winooski, VT, USA).

### 2.12 Statistical analysis

Data are shown as the mean ±SD deviation, and all experiments were performed in triplicate, each being repeated at least three times. Data of cell proliferation were analyzed via one-way ANOVAs, and cell cycle distribution, cell apoptosis, levels of ROS and *GPX5*, and activity levels of SOD, CAT, and GSH-Px were compared using the independent sample t test in SPSS software (SPSS Inc., Chicago, IL, USA).

## 3 Results

### 3.1 Morphology and identification of sheep EECs

The primary epididymal cells obtained by enzymatic digestion were mostly cell clusters with only a small number of single cells (Fig. 1a). After one passage, the culture contained a large number of single cells with very few fibroblasts. The monolayer cells reached confluence after 2–3 d of culture (Fig. 1b). To detect whether the epithelial marker, cytokeratin 18, can be expressed in isolated purified EECs, an IF assay was performed using mouse monoclonal antibody targeting cytokeratin 18. Immunofluorescence showed that isolated EECs could be reactive with cytokeratin-18-specific antibodies, whereas fibroblasts as a control did not express keratin 18 (Fig. 2). Thereafter, the isolated epithelial cells were used for later experiments.

### 3.2 Trehalose effectively improves proliferation viability of sheep EECs

As shown in Fig. 3a, the exponential growth period of cells was during days 2–4. The proliferation of cells in the 100 mM trehalose group was significantly higher than that of the control group on days 2–3 (P<0.05) and on days 4–5 (P<0.01). The growth activity of cells in 50 mM trehalose group on day 3–4 was significantly higher than that of the control group (P<0.05). There was no significant difference in the growth activity of cells between the 200 mM trehalose group and the control group (P>0.05).

EECs cultured in vitro with 100 mM trehalose could be subcultured stably for 14 passages, while cells cultured without trehalose could only be subcultured for 5 passages (data not shown). To test whether the cells cultured with 100 mM trehalose could maintain epididymal characteristics, we investigated the presence of the epididymal molecular markers *GPX5* and *AR* by RT-PCR. As shown in Fig. 3b, these markers were detected in EECs cultured with trehalose at passages 0, 1, 6, and 14 and EECs without trehalose at passage 0, indicating that subcultured EECs with 100 mM trehalose could maintain epididymal functional characteristics.

### 3.3 Trehalose alters the cell cycle distribution and apoptosis of sheep EECs

We investigated cell cycle distribution after treating cells with a medium containing 100 mM trehalose. Cells cultured with 0 mM trehalose were used as a control. Flow cytometry analysis showed that, compared with the control group, the number of cells cultured with 100 mM trehalose in S phase was significantly increased (P<0.01), while the number of cells in G1 phase was significantly decreased (P<0.01), and there was no significant difference in G2 phase cells (P>0.05, Fig. 4a), which further indicated that trehalose could effectively improve the cell proliferation activity.

Furthermore, we also investigated cell apoptosis after treating cells with a medium containing 100 mM trehalose. The results of flow cytometry analysis indicated that the apoptotic index of EECs treated with trehalose (100 mM) was significantly lower than that of untreated EECs (P<0.01, Fig. 4b), which suggested that trehalose could effectively inhibit the apoptosis of EECs.

### 3.4 Antioxidant effect of trehalose in sheep EECs

The quantification of ROS and the activities of superoxide dismutase (SOD), catalase (CAT), and glutathione peroxidase (GSH-Px) in cells cultured with 0 or 100 mM trehalose was measured. Quantitative fluorescence measurements are summarized in Fig. 5a, in which treatment with 100 mM trehalose led to a robust decrease in ROS (dichlorofluorescein (DCF) fluorescence) of EECs compared with the control (P<0.01). Furthermore, antioxidant detection in the 100 mM trehalose treatment group compared with the trehalose-free treatment group (Fig. 5b) showed no significant change in SOD activity (P>0.05), whereas the 100 mM trehalose treatment group had higher CAT activity (P<0.01) and GSH-Px activity (P<0.05) in EECs.

Regulation of *GPX5* by trehalose is demonstrated in Fig. 5c. The expression of *GPX5* mRNA in the 100 mM trehalose treatment group was significantly higher than that in the control group (P<0.01). Furthermore, the expression of GPX5 protein under trehalose treatment is shown in Fig. 5d. GPX5 protein was significantly upregulated in the 100 mM trehalose treatment group compared with the control group (P<0.05).

## 4 Discussion

Trehalose is a nonpermeable cryoprotectant that can improve the membrane integrity of mammalian cells during cryopreservation (Ateşşahin et al., 2008; Saxena et al., 2012; Ha et al., 2016; Kamalifar et al., 2018). In the present study, trehalose had a concentration-dependent effect on EEC proliferation. We determined that 100 mM trehalose was the optimum concentration and enabled EECs to be serially passaged 14 times with key molecular markers expressing normally, indicating that trehalose can improve the proliferation of EECs in vitro and maintain their normal functional characteristics. Furthermore, 100 mM trehalose significantly increased the proportion of S-phase cells while reducing the proportion in G1 phase, and it reduced the apoptotic index of cells. Similar results were reported in mouse epithelial cells (Qu et al., 2014). Together, these results showed that 100 mM trehalose could effectively improve the growth activity of EECs in vitro. It is noteworthy that there was no significant difference in the growth activity of cells between the 200 mM trehalose group and the control group. This may result from the following: when the extracellular trehalose concentration exceeds the saturation level, the cell is stressed by the excess dehydration due to the remaining osmotic pressure, with apoptosis occurring (Uchida et al., 2019).

Studies have shown that trehalose plays a role in protecting cells against oxidative stress (Nishimoto et al., 2016). SOD, CAT, and GSH-Px, as important components of the enzymatic antioxidant system, protect the cell from oxidative stress (Bucak et al., 2007). In this study, treatment with 100 mM trehalose significantly decreased ROS levels and increased the activity of CAT and GSH-Px in EECs. These results suggest that trehalose may scavenge oxygen free radicals by stimulating antioxidants in EECs and protect cells from oxidative stress. Our results are consistent with previous reports demonstrating that trehalose can significantly increase the CAT content of diluted semen after freezing and thawing and improve semen quality (Bucak et al., 2007; Hu et al., 2010).

GPX5, a member of the glutathione peroxidase family, is a selenium-free antioxidant enzyme highly expressed in the cytoplasm and static cilium of caput epididymis epithelial cells (Koziorowska-Gilun, 2013; Li et al., 2018). In addition, GPX5 can be secreted into epididymal fluid and seminal plasma to compensate for the congenital loss of antioxidant enzymes in immature sperm (Maňásková-Postlerová et al., 2011). The expression of *GPX5* in epididymis is higher than that of other members of glutathione peroxidase family (Brigelius-Flohé, 2013). Interestingly, our study found that 100 mM trehalose effectively increased the mRNA and protein expression of *GPX5* in sheep EECs, indicating that trehalose played an antioxidant role in sheep EECs by regulating the expression of *GPX5*. However, the detailed mechanism of trehalose-regulated *GPX5* expression in sheep epididymis needs further investigation.

## 5 Conclusions

In a summary, the present study indicated that trehalose can effectively protect EECs from oxidative stress by increasing the activity of CAT and GSH-Px and upregulating *GPX5* expression. It could be employed to maintain vitality of sheep EECs during long-term in vitro culture.

## Data Availability

The data are available from the corresponding author upon request.
